# Spray-Dried Structured Lipid Carriers for the Loading of *Rosmarinus officinalis*: New Nutraceutical and Food Preservative

**DOI:** 10.3390/foods9081110

**Published:** 2020-08-13

**Authors:** Iara Baldim, Claudia R. F. Souza, Alessandra Durazzo, Massimo Lucarini, Antonello Santini, Eliana B. Souto, Wanderley P. Oliveira

**Affiliations:** 1 Faculty of Pharmaceutical Sciences of Ribeirão Preto, University of São Paulo, Av. do Café s/n, Ribeirão Preto, SP, São Paulo 14040-903, Brazil; iara.baldim@usp.br (I.B.); souzacrf@gmail.com (C.R.F.S.); 2 CEB–Centre of Biological Engineering, University of Minho, Campus de Gualtar, 4710-057 Braga, Portugal; 3 CREA-Research Centre for Food and Nutrition, Via Ardeatina 546, 00178 Rome, Italy; alessandra.durazzo@crea.gov.it (A.D.); massimo.lucarini@crea.gov.it (M.L.); 4 Department of Pharmacy, University of Napoli Federico II, Via D. Montesano 49, 80131 Napoli, Italy; 5 Faculty of Pharmacy, Department of Pharmaceutical Technology, University of Coimbra, Pólo das Ciências da Saúde, Azinhaga de Santa Comba, 3000-548 Coimbra, Portugal

**Keywords:** *Rosmarinus officinalis*, antioxidant activity, spray-dryer, structured lipid carriers, food preservative, nutraceuticals

## Abstract

Rosemary, an aromatic herb with significant antioxidative activity, is frequently used as food preservative and a source of nutraceuticals. Its antioxidant effect is mainly related to the presence of phenolic compounds, molecules considerably unstable and prone to irreversible physicochemical changes when exposed to external agents. We here proposed the loading of rosemary into structured lipid systems to improve its physicochemical properties. Four formulations were prepared using the same amount of rosemary lyophilized extract. The lipid phase was composed of stearic acid and oleic acid, and the aqueous phase, a varying combination of drying carriers (whey protein concentrate or gum Arabic) and surfactant (Poloxamer 188). The formulations were sonicated, spray-dried, and the obtained powders were characterized regarding the density (0.18 g/mL to 0.26 g/mL), particle size distribution (7 µm and 52 µm), and water solubility (29% to 48%). The antioxidant activity was determined by applying ABTS^•+^ radical-scavenging assay and the results expressed per gram of lyophilized extract (150.6 μmol Trolox/g to 376.4 μmol Trolox/g), with a significantly lower/higher result seen for formulations containing gum Arabic and a higher concentration of Poloxamer. The prepared systems may have potential applications as preservative in foodstuff and as nutraceutical.

## 1. Introduction

There has been an increasing interest in the use of natural antioxidants [1,2,3,4] to replace the synthetic ones [5,6,7,8,9,10]. This trend is motivated by some negative aspects of the synthetic antioxidants, such as some deleterious secondary effects [11] and the concern about their toxicological long-term effects [12]. Studies have also demonstrated that butylated hydroxyanisole (BHA) and butylated hydroxytoluene (BHT), the two most commonly employed antioxidants in the industry, are associated with DNA damage induction [13,14].

Herbs, spices, and essential oils are well known to possess antioxidant activity [15,16,17,18,19,20,21,22]. *Rosmarinus officinalis* is a common household plant [23], with significant antioxidant properties [24,25], being broadly applied in various fields, including cosmetics [26], as food preservative [27], as nutraceutical, and in phytomedicines [16,28,29]. These properties have been attributed to rosemary’s polyphenolic composition, which includes mainly rosmarinic acid [30], carnosic acid, and carnosol [31].

The antioxidant properties are the expression of combined action of bioactive components in a matrix [32]. Several methodologies are commonly used for this evaluation [33], such as the Trolox equivalent antioxidant capacity (TEAC), the total radical-trapping antioxidant parameter (TRAP), the oxygen radical absorbance capacity (ORAC), the ferric-reducing/antioxidant power (FRAP), and the 2,2-diphenyl-1-picrylhydrazyl radical (DPPH^•^) scavenging method [9,10]. Each essay has its peculiarities of mechanism and methodology to evaluate the antioxidant properties; therefore, it is recommended to use different tests for an adequate analysis.

Herbs are routinely marketed as standardized products (of known bioactive content) under different dosage forms. Recently, the use of standardized dried extracts is a trend of the industry. Encapsulation of phytochemicals in drug delivery systems is a suitable way to improve their physical-chemical properties, prevent their degradation, and also to improve their bioavailability in biological settings [34,35]. The highly lipophilic nature of rosemary antioxidants makes encapsulation in lipid systems the most suitable. The solid form has clear advantages over the conventional liquid form, namely: higher physicochemical and microbiological stability; ease of standardization; a higher bioactive content; ease in logistics processes; and the possibility to be used under distinct dosage forms [34,36]. The spray-drying, one of the most used drying techniques, is widely used to standardize phytopharmaceutical ingredients, ensuring product efficacy, safety, and quality [37]. It is also a widely used approach for the microencapsulation of compounds of different chemical nature, producing microspheres or microcapsules, according to the initial feed material [34,38,39].

The selection of the appropriated drying carriers is a key parameter to obtain a stable product [40]. The composition of the feed formulation directly influences the spray-dried product recovery since it affects the glass transition temperature of the composition [41]. Carbohydrates are examples of widely used wall material to encapsulate food ingredients by spray-drying [42,43,44]. On the one hand, these materials offer several desirable properties for encapsulating agents, such as good protection of flavors against oxidation, low viscosity at high solids contents (such as cellulose) [45], good solubility in water, versatility, and GRAS (generally recognized as safe) status [46]. In contrast, most lack the interfacial properties, important characteristics associated with some encapsulation materials (e.g., milk proteins) [47]. Nevertheless, since they have a higher cost than carbohydrates, it becomes disadvantageous to use exclusively proteins for the encapsulation process. An interesting approach to improve the encapsulating properties of a system is the use of blends of carbohydrates and proteins, combining the advantages of both ingredients [48]. Gum Arabic is another effective wall material considering its emulsifying properties due to the protein portion of its structure [49].

It is also noteworthy that pharmaceutical and food lipid formulations prioritize the use of natural components [15,20,50,51,52]. Meanwhile, the possibility of using generally recognized as safe and natural ingredients expands the applicability of lipid nanoparticles not only in the delivery of drugs but also in several other fields and market segments [50,51,53,54]. The solid lipid matrix can be composed of waxes, fatty acids, glycerides, and steroids. Stearic acid, a long-chain saturated fatty acid, is a constituent of animal fats. Properties such as melting point above room and body temperatures, good biocompatibility, and reduced toxicity make this a widely used pharmaceutical excipient for drug delivery [52,55]. The presence of a liquid lipid (oil) reduces the melting point of solid lipid and creates imperfections in the lipid matrix, which increases the loading capacity, especially for lipophilic compounds [53,54,56]. Oleic acid is a fatty acid found in several animal and plant sources [57], commonly used as an emulsifying agent and penetration enhancer [53].

This study aimed to produce powdered dried solid lipid dispersions containing *Rosmarinus officinalis* lyophilized extract and evaluate the influence of drying adjuvants (whey protein and Arabic gum) and surfactant concentration (Poloxamer 188) on the spray-drying process, physicochemical characteristics of the powder, and antioxidant properties of the dried product.

## 2. Materials and Methods

### 2.1. Materials

6-hydroxyl-2,5,7,8-tetra methylchroman-2-carboxylic acid (Trolox), ABTS, dehydrated quercetin and gallic acid were purchased from Sigma–Aldrich Chemical Co (St Louis, MO, USA); ethanol and potassium per-sulphate were purchased from LabSynth (Diadema, SP, Brazil); *Rosmarinus officinalis* leaves (Santos Flora, São Paulo, Brazil), stearic acid (Viafarma, São Paulo, Brazil), oleic acid (LabSynth, São Paulo, Brazil), Poloxamer 188 (kindly donated by BASF, Brazil), Arabic gum (NEXIRA, Brazil), whey protein (Arla Foods Ingredients S.A., Argentina), ethanol (LabSynth, Diadema, SP, Brazil), and ultrapure water was obtained by Milli Q filtration system (Millipore, Germany).

### 2.2. Preparation of Lyophilized R. Officinalis Extract

Hydroalcoholic extract of milled leaves of *Rosmarinus officinalis* was prepared by dynamic maceration using ethanol 70% *v*/*v* as the solvent and a plant to solvent ratio of 1:10 (*w*/*v*). The glass container was coupled to a thermostatic bath at 50 °C for 60 min. At the end of the process, the extract was vacuum filtered and concentrated in a rotary evaporator (55 °C and 600 mmHg). The concentrated extract was subsequently lyophilized in a Thermo Fisher Micromodulyo-115.

### 2.3. Preparation of Lipid Formulations

Based on the components’ proportions and process conditions obtained in previous preformulation studies conducted by our research group, with some modifications [41], four lipid formulations were prepared in a single trial, containing the same amount of rosemary lyophilized extract (Table 1). The oil phase (stearic acid and oleic acid) was heated to 75 °C to melt the lipid mixture. The aqueous phase, composed by water, rosemary extract solubilized in ethanol, drying carrier (whey protein or gum Arabic, depending on the formulation), and surfactant (Poloxamer 188), was heated at the same temperature (75 °C), and slowly added to the oil phase. The formed preformulation was then sonicated in an ultrasonic sonicator VCX750 (SONICS Vibracell, Newton, PA, USA), using a 13 mm probe under 20 kHz of frequency and intensity of 50% for 5 min, prior to the drying.

### 2.4. Drying of Lipid Compositions

The lipid dispersions were dried in a bench-top SD-05 spray-dryer (Lab-Plant Ltd., Filey, North Yourkshire, UK) and the spray-drying conditions were based on Cortés-Rojas et al. with some modifications [41]. Operating conditions were set as the feed flow rate of 4 g/min, through an atomizer with an orifice of 0.5 mm, inlet gas temperature at 90 °C, drying gas flow rate 60 m^3^/h, pressure and spray gas flow of 3 kgf/cm^2^ and 17 Lpm, respectively.

### 2.5. Moisture Content and Water Activity

For the determination of moisture content, an amount of 100 mg of powder was taken in a Karl Fischer 870 Titrino Plus Methrom (Herisau, Switzerland), immediately after spray-drying. Water activity was measured in an Aqua Lab 4Tev^®^ equipment (Decagon devices, Pullman, WA, USA) using the dew point sensor. Results are results expressed as the mean and standard deviation of triplicate measurements [58].

### 2.6. Spray-Dried Product Recovery

The efficiency of the spray-drying process was evaluated through the determination of product recovery (R). This parameter, defined as the ratio between the total amount of powder recovered after drying and the mass of formulation fed to the system, was evaluated using a mass balance according to Equation (1).

(1) $$R(\%)=\frac{M_{c}\times (1-X_{p})}{W_{s}\times C_{s}\times \theta }\times 100$$

where *Mc*, the mass of collected powder (g); *Xp*, powder moisture content (g); *Ws*, liquid formulation feed rate (g/min); *Cs*, solid content of the feed liquid formulation (g/g); and *θ*, process time (min).

### 2.7. Analytical Evaluation of Flow Properties

Flowability of powders was determined by calculating the Hausner Ratio (HR) and Carr’s Index (CI), from the bulk density (*d*_0_, where *d*_0_ = m_0_/V_0_) and tapped bulk density (*d*_1250_, where *d*_1250_ is powder density after tapping the probe 1250 times), according to Equations (2) and (3).

(2) $$HR=\frac{d_{1250}}{d_{0}}$$

(3) $$CI=\frac{d_{1250}-d_{0}}{d_{1250}}\times 100$$

The analysis was performed in a Caleva^®^ Tapped Density Tester Type TDT (Frankfurt, Germany), with a distance of 14.0 mm for the tapping probe [27].

### 2.8. Particle Size and Particle Distribution

To evaluate the powder particle size, an aliquot of dried samples was dissolved in ultrapure water at a ratio of 1:25 (p/v) and the analysis was performed by laser diffractometry in a Light Scattering Beckman Coulter LS 13,320 (Brea, CA, USA), equipped with a liquid module for determination of the particle size range between 0.017 µm and 2000 µm. The obtained data were evaluated using volume distribution. After dilutions, the samples were subjected to mechanical stirring for 20 min and subsequently processed in the ultrasonic probe Vibracell (model VC750-SONICS, Newtown, PA, USA) with the microtip probe 1/8”, at 20 kHz of frequency and 30% of intensity for 30 s. An optical microscope (Olympus-model BX60MIV, Tokyo, Japan) coupled to an image analyzer software (Image-Pro Plus 4.5, Media Cybernetics Inc., Bethesda, Rockville, MD, USA) was used for determination of the particle distribution of the microcapsules. The images were obtained with an increase of 50 and 500 times. The number of particles was counted according to the mean particle diameter of 0 to 200 µm.

### 2.9. Water Solubility Study

Evaluation of water solubility of powders was performed as described by Cano-Chauca et al. with some modifications [59]. Shortly, aliquots of 0.5 g of each powder were precisely weighed and dissolved in water (50 mL) before the homogenization under magnetic stirring for 10 min at 900 rpm. Afterward, the samples were centrifuged (Eppendorf model 5430 R, Eppendorf, Hamburg, Germany) at 2330 g for 5 min and an aliquot of the supernatant (15 mL) was withdrawn and placed in a tared Petri dish. The total solid content was determined after 5 h of oven drying at 105 °C.

### 2.10. Particle Morphology of Microcapsules

Scanning electron microscopy with field emission (Inspect F-50, FEI, Eindhoven, Nederland) was performed to visualize the morphological aspects of particles. Samples were mounted onto stubs with double-sided adhesive tape and a thin layer of platinum was applied to the surface of the particles to increase the conductivity and the analysis was made at 5 kV.

### 2.11. Antioxidant Activity

The antioxidant capacity analysis was performed in an Agilent 8453 diode array UV-Visible spectrophotometer (Agilent Life Sciences and Chemical Analysis, Santa Clara, CA, USA), as described by [60]. Briefly, ABTS radical cation (ABTS^•+^) was produced by reacting 7 mM ABTS stock solution and 2.45 mM potassium persulphate after incubation in the dark, at room temperature during 16 h. The ABTS^•+^ solution was diluted with ethanol to reach an absorbance of 0.700 ± 0.020 at 734 nm. After the proper dilution of the sample (ethanol mixed with 3 mL of diluted ABTS^•+^ solution), the absorbance reading was taken at 734 nm exactly 1 min after initial mixing and up to 6 min. Ethanol was used as blank and standard solutions in concentrations from 0 to 19.8 µm were used for the construction of the Trolox calibration curve. The concentration of Trolox for the standard reference solution was used to calculate the percentage of inhibition of absorbance at 734 nm, plotted as a function of Trolox concentration for the standard reference data. The absorbance of the calibrated Trolox standard solution was compared to the resulting oxidized solution. Results were expressed as µmol Trolox equivalents/g of plant extract (TEAC) using the calibration curve of Trolox.

### 2.12. Statistical Analysis

Statistical analysis was carried out using one-way analysis of variance (ANOVA) with Tukey post hoc test to identify significant differences between means (*p* < 0.05), employing the software GraphPad^®^ Prism 8.

## 3. Results and Discussion

**Figure 1:**
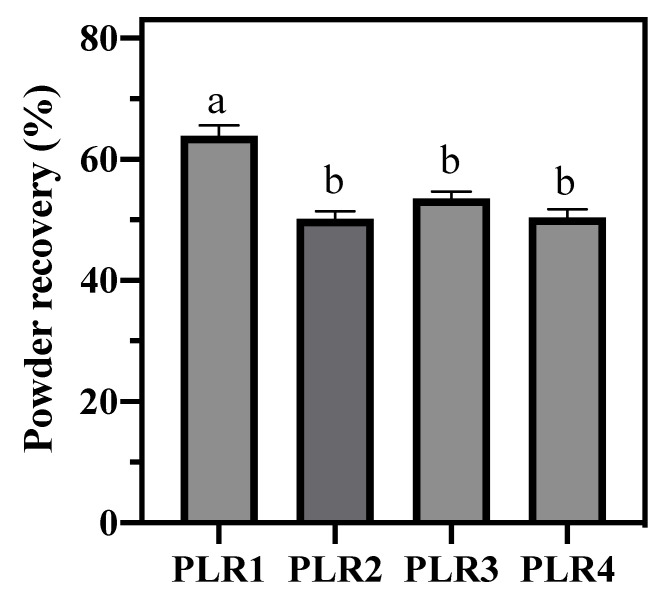
Powder recovery of spray-dried lipid dispersions (*n* = 3). Same letter means no significant difference according to Tukey’s multiple comparison test (*p* < 0.05).

**Figure 2:**
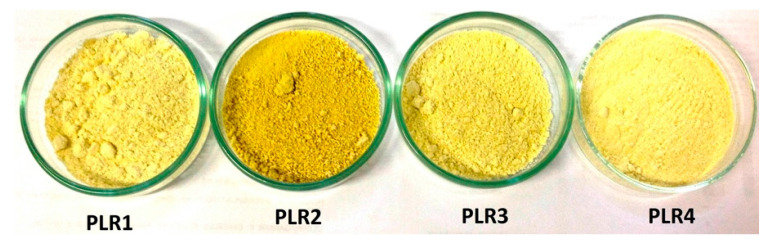
Appearance of spray-dried formulations.

To obtain information about the impact of the composition on the spray-drying performance, we determined the product recoveries (R) during the spray-drying process of four different formulations (PLR1, PLR2, PLR3, and PLR4), as represented in Figure 1. Three drying processes were performed for each formulation. The association of whey protein and the low concentration of Poloxamer (PLR1) resulted in the highest powder recovery among the evaluated samples. Notwithstanding, this formulation presented high viscosity, which made it difficult to atomize in spray-drying. In this way, it was heated at 45 °C during the atomization process, to avoid the blockage of the atomizing system; this step might have influenced the process yield. Except for PLR1, the formulations presented a very similar drying yield. Powdered products showed similar macroscopic appearance, except for PLR2 formulation, which presented a darker, hygroscopic, and granular powder (Figure 2).

### 3.1. Solubility Test

**Figure 3:**
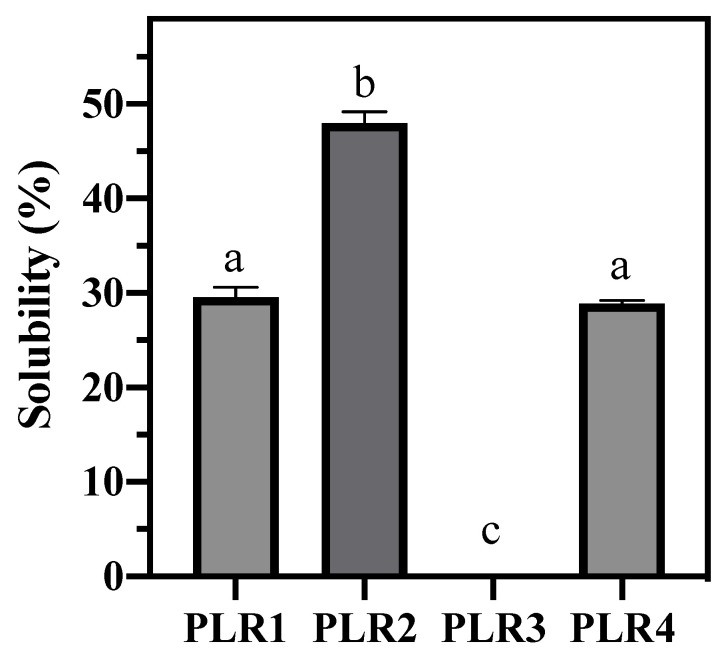
Water solubility of spray-dried powders. Same letter means no significant difference according to Tukey’s multiple comparison test (*p* < 0.05).

The aqueous solubility of spray-dried samples is shown in Figure 3, whose analyzed samples were obtained by a single drying process for each formulation. The results showed that the formulation containing whey protein and a high concentration of Poloxamer (PLR2) is the most water-soluble sample among those analyzed. Poloxamers, a class of water-soluble nonionic surfactants, are block copolymers of polyoxyethylene-polypropylene with an amphiphilic character. These polymers find use in many applications that require a wetting or solubilizing agent and surface adsorption excipients. They are frequently employed as excipients for pharmaceuticals to facilitate solubilization, dissolution, and bioavailability of poorly water-soluble compounds [53]. It was observed, as expected, that a high concentration of Poloxamer increases the water solubility of the formulations. The association of gum Arabic and low concentration of Poloxamer (PLR3) resulted in a powder insoluble in water. Some previous literature reports have indicated a correlation between the surface morphology of the particles and the instant powder solubility [59,61]. Highly ordered particles generally present lower solubility. Gombas et al. (2003) reported that crystalline and amorphous particles also differ physicochemically concerning the size and shape of the particles, solubility in water, hygroscopicity, and flow properties. Despite that we have not directly determined the crystallinity of the obtained particles, the flow properties (poor flow) and the morphology of particles (higher degree of uniformity regarding the rounded shape) presented by PLR3 particles endorses its insolubility in comparison to other formulations. Regarding the drying carriers, the results pointed to considerably higher water solubility for the formulations containing whey protein (PLR1 and PLR2) when compared to those containing gum Arabic (PLR3 and PLR4).

### 3.2. Powder Characterization and Flow Properties

The powder properties, present in Table 2 and Table 3, show a small variation in particle size, which was within the expected range for spray-dried powders [58]. The size distribution was calculated based in the particle size in the percentages of 10%, 50% and 90%, and the span, defined as presented in Equation (4), where dx is a particle size value such that, in a cumulative distribution, x% of the particles have a smaller size [62]. It can be observed that the span values were very similar for the formulations with whey protein, the same occurs for formulations containing gum Arabic in the composition, which indicates similar polydispersity between the formulations with the same drying carrier. The high standard deviation found for the particles is justified by the method of analysis used. The values are expressed in volume, which biased the result with the presence of a small number of larger particles, as is the case. Our raw data demonstrate high values of d_100_, in the range of a few tens of µm (data not shown), which influences the standard deviation values.

(4) $$Span=\frac{d_{90}-d_{10}}{d_{50}}$$

The flow properties were determined based on tapped and bulk powders densities, which were calculated by the Hausner Ratio (HR) and Carr Index (CI). Both of these factors are related to particle flowability and are based on characteristics such as friction and accommodation of particles, factors that in turn are affected by the size, shape, and surface characteristics of the particles. The greater values indicate high cohesiveness and therefore, weak flow [41,63]. Hausner ratio values above 1.2 characterize a good flow. Carr index classifies the flowability of the powders according to intervals [64]:

1. Between 5% and 15%: excellent flow
2. Between 12% to 16%: good flow
3. Between 18% to 21%: scarce flow
4. Between 23% to 35%: poor flow
5. Between 33% to 38%: very poor flow
6. Higher than 40%: extremely poor flow

Incorporation of a higher concentration of Poloxamer has been shown to reduce the cohesiveness and improve the flowability of the spray-dried powders, once both PLR2 and PLR4 exhibit excellent and good flow respectively (Table 3). Nevertheless, both formulations containing a smaller amount of Poloxamer (PLR1 and PLR3) presented scarce and weak flow. The flowability of the powders was also dependent on the type of drying carrier used. Formulations containing whey protein (PLR1/PLR2) provided better flowable powders. The higher particle size distribution (d_90_) presented by the samples containing whey protein, when compared to the corresponding samples with Arabic gum, support this result. The presence of whey protein concentrate in the feed material increases its viscosity, which results in a powder with bigger particles [65]. Furthermore, it is well known that mean particle size, shape, and particle size distribution are characteristics strictly related to the flow properties [66]. The increase in mean particle size tends to reduce the cohesiveness between the powder particles, which results in increased flowability [65].

Other important parameters to consider when characterizing powders are moisture content and water activity. PLR1 and PLR2 powders presented higher values for both moisture content and water activity. This can be attributed to the fact that proteins in an amorphous state hold an increased capacity to incorporate water molecules, which increases the moisture content and, indirectly, the water activity. Our findings are in agreement with a previous study from Bhusari et al. (2014) comparing different carrier agents to investigate their effect on the physicochemical properties of spray-dried powders [65].

In general, the limited flowability presented by the powders is normally obtained for spray-dried powders and corroborate with results previously found by our research group [67]. These findings are closely related to the stickiness of the lipid formulations to the drying chamber of the equipment [41]. The high temperature used for the spray-drying process, even for a short time, is sufficient to melt the lipid components of the formulations, favoring the powder adherence to the walls of the drying chamber and the cyclone. The drying carriers assist in this process by increasing the glass transition temperature of the lipids and improving the powder flow by increasing its density [37,41,64].

**Figure 4:**
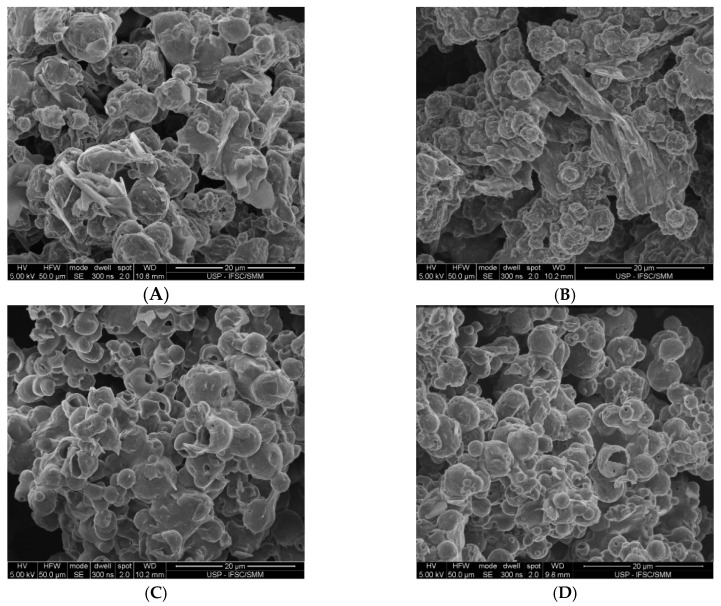
Particle morphology of spray-dried lipid carriers: PLR1 (**A**), PLR2 (**B**), PLR3 (**C**) and PLR4 (**D**).

Figure 4 represents the particle morphology of lipid-based powders. The morphology of the spray-dried particles is directly related to drying conditions and the constituents of the encapsulating composition [41]. The physicochemical properties of the material to be atomized in the spray-drying partially determine the drying behavior and particle morphology of the final product [68]. The powders showed particles in different sizes, confirming the results obtained for particle size distribution. Surface analysis of the particles revealed a rounded shape with a rough and shriveled external surface, typical of spray-dried powders [69]. The shriveled and rough surfaces were more pronounced for the powders containing whey protein in the composition (A and B). These findings can be related to the protein fraction and agree with the findings of Bhusari et al. (2014) [65] and Faldt and Bergenstahl (1994) [70] that related the shriveled surface of the particles to the characteristic of protein-rich spray-dried powders. Furthermore, Anandharamakrishnan et al. [71] found that whey protein solution spray-dried at high temperature (100–120 °C) presented irregularly shaped particles, which confirms that mild temperatures, around 60–80 °C, are suitable to avoid protein denaturation. Particles of regular surface are usually preferred as they contribute to improving the flowing properties of the powders.

### 3.3. Antioxidant Properties of Lipid Carriers

**Figure 5:**
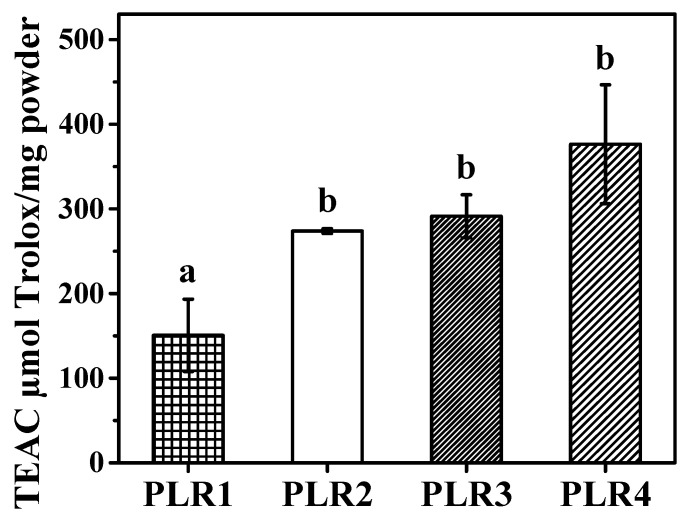
Antioxidant profile of spray-dried lipid carriers expressed as Trolox equivalents per mg of powder. Same letter means significantly different in Tukey’s multiple comparison test (*p* < 0.05).

The in vitro antioxidant profile of the spray-dried lipid carriers was measured right after preparation and the results are presented in Figure 5. The non-encapsulated lyophilized extract, as expected, presented a very high antioxidant activity (1356 ± 91 µmol Trolox/mg powder—data not shown). The differences between the compositions of the lipid systems provided distinct values for antioxidant activity, which may affect the release of the antioxidant molecules to the reaction medium. At *p* < 0.05 the differences between the formulations PLR2, PLR3, and PLR4 were not statistically significant. On the other hand, all of these formulations showed antioxidant activity statistically superior to PLR1, which demonstrates that the combination of whey protein and low concentration of surfactant (Poloxamer) resulted in a product with reduced antioxidant activity. Our results are in good agreement with the research findings reported by the different researchers [68,69,70], which reported the increment in the antioxidant activity by increasing the concentration of antioxidants molecules into the aqueous phase of emulsions by surfactant micelles. This mechanism is related to the ability of surfactant micelles in solubilizing the antioxidant molecules out of the emulsion droplet core, where the molecule is inefficient as an antioxidant [72].

## 4. Conclusions

Powdered redispersible lipid-based compositions entrapping antioxidants from *Rosmarinus officinalis* extract were successfully generated by spray-drying. Our study has been a preliminary assessment on the evaluation of the influence of selected drying adjuvants (whey protein and gum Arabic) and surfactant concentration (Poloxamer 188) on the spray-drying process, powder properties, and antioxidant profile of the dried lipid carriers. Both the surfactant concentration and type of drying adjuvants influenced the physical properties and antioxidant activity of the final product. The association of whey protein and the low concentration of poloxamer resulted in a highly viscous formulation, difficult to be atomized in the spray-drying. Despite similar particle size and polydispersity between the powders, the formulation containing a higher concentration of poloxamer and whey protein showed the best flow properties; however, morphologically formulations with gum Arabic showed homogeneously rounded particles. The spray-dried product exhibited high antioxidant activity, mainly the formulations containing gum Arabic and higher poloxamer concentration, anticipating its potential use as a natural preservative in foodstuff and as a nutraceutical. Besides, recognizing that selected adjuvants do influence the outcomes of the production, a full factorial design experiment can be proposed to describe the best conditions to achieve the highest product recovery.

## Figures and Tables

**Table 1:** Composition of the lipid systems containing *R. officinalis* lyophilized extract (% *w*/*w*).

Component	Function	Formulation			
		PLR1 *	PLR2 **	PLR3 *	PLR4 **
Stearic acid	Solid lipid	9	9	9	9
Oleic acid	Liquid lipid	1	1	1	1
Whey Protein	Drying carrier	5	5	-	-
Gum Arabic		-	-	5	5
Rosemary extract	Antioxidants source	2	2	2	2
Ethanol	Solvent	5	5	5	5
Water		78	78	78	78

* Containing the surfactant Poloxamer 188 in the concentration of 10% of the amount of lipid; ** Containing the surfactant Poloxamer 188 in the concentration of 50% of the amount of lipid.

**Table 2:** Laser diffractometry (LD) diameters distribution of spray-dried lipid dispersions.

Form	Particle Size (µm)	LD (µm)		
		d_10_	d_50_	d_90_
PLR1	0.764 ± 0.627	0.43	0.61	1.17
PLR2	0.747 ± 0.609	0.43	0.61	1.16
PLR3	0.759 ± 0.612	0.43	0.61	1.19
PLR4	0.772 ± 0.631	0.43	0.62	1.22

**Table 3:** Powder characterization (optical micrography) and flow properties of spray-dried lipid dispersions.

Form	Particle Distribution (µm)				Hausner Ratio (-)	Carr Index (%)	Moisture Content (%)	Water Activity (-)
	d_10_	d_50_	d_90_	Span (-)				
PLR1	1.28	4.95	12.77	2.37	1.2	20.0	2.94 ± 0.11	0.398 ± 0.016
PLR2	2.07	6.07	15.69	2.24	1.1	6.1	2.79 ± 0.29	0.447 ± 0.005
PLR3	2.26	5.85	12.69	1.78	1.2	25.0	2.32 ± 0.25	0.291 ± 0.056
PLR4	2.09	6.17	14.32	1.98	1.2	16.7	2.24 ± 0.01	0.259 ± 0.031
